# Interventions in Acute or Subacute Phase for Type B Aortic Dissection

**DOI:** 10.3400/avd.ra.24-00012

**Published:** 2024-03-26

**Authors:** Masaaki Kato

**Affiliations:** 1 Department of Cardiovascular Surgery, Morinomiya Hospital, Osaka, Osaka, Japan

**Keywords:** type B aortic dissection, TEVAR, complicated, uncomplicated high-risk feature

## Abstract

The treatment strategy for acute and subacute Stanford type B aortic dissection has changed significantly since the advent of thoracic endovascular aortic repair (TEVAR). Indication for invasive treatment: In addition to the conventional complicated cases (rupture or malperfusion case), the indication for invasive treatment now includes cases with refractory hypertension, persistent or recurrent pain, large aortic diameter, and other conditions that are considered to have a poor prognosis with conservative treatment. Treatment methods: TEVAR is the first choice for acute, subacute, and early chronic-stage treatment, and when this is not possible, other techniques (fenestration and graft replacement) are chosen. Treatment timing: The timing of invasive treatment should be emergent in life-threatening conditions (for rupture or malperfusion case) and immediate in symptomatic cases, while in other cases, preemptive TEVAR is considered appropriate on a scheduled timing within 6 months of onset. (This is a translation of Jpn J Vasc Surg 2023; 32: 157–163.)

## Introduction

The introduction of thoracic endovascular aortic repair (TEVAR) for Stanford type B aortic dissection (type B dissection) since 2000 has revolutionized the treatment strategy for type B dissection. The number of invasive treatment cases with type B dissection increased, especially in the acute and subacute phases. Of course, this is due to the fact that the indications for invasive treatment themselves have changed significantly with the widespread of TEVAR. This article describes the changes in treatment strategies for the acute and subacute phases of type B dissociation, contrasting the indications and methods used before 2000 with those used today.

## Changes in Indications for Invasive Treatment

Prior to 2000, the basic strategy for acute type B aortic dissection was antihypertensive treatment and bed rest for uncomplicated cases except for ruptures and malperfusions. On the other hand, emergency surgery was indicated if a complicated (rupture or malperfusion) type B dissection was diagnosed. However, the results of emergency surgery for ruptured cases were extremely poor (surgical mortality rates of 21%–92%), and surgical outcomes for malperfusion cases with intestinal ischemia were as poor (surgical mortality rate of 43%) as those for ruptured cases.^1^^–^^4)^

The indications for invasive treatment of these ruptures and malperfusions have not changed at all since 2000, but the main treatment modality has changed to emergency TEVAR. The major change in the indication for invasive treatment of acute and subacute type B dissections since 2000 has been in the treatment strategy for “complicated cases” other than rupture and malperfusion that occur in the acute phase (within 48 hours of onset), that is “cases with a poor prognosis with continued medical conservative treatment” such as uncontrolled hypertension, persistent or recurrent pain, and large aortic diameter.^5^^–^^7)^ Since the advent of TEVAR, the results of invasive treatment for these newly added broadly defined complicated cases have improved dramatically^8^^–^^12)^ and have surpassed those of continued conservative treatment, so they are now included as an indication for invasive treatment.

**Table 1** shows the current indications for invasive treatment of acute and subacute type B dissections, or complicated cases in the broad sense, compared to those prior to 2000.

**Table:** Table 1 Indications of intervention for acute/subacute type B aortic dissection

Type of complication	IRAD name	Name in SVS/STS reporting standard	Current invasive treatment	Invasive treatment before 2000
Rupture (impending rupture)	Complicated case	Complicated case	TEVAR	Graft replacement
			When TEVAR is unsuitable, graft replacement	
Malperfusion			TEVAR	Fenestration (EVT or open)
			Fenestration (EVT or open)	Direct intervention on the responsible vessel (EVT or bypass)
			Direct intervention on the responsible vessel (EVT or bypass)	Bypass
Refractory hypertension		Uncomplicated high-risk feature	TEVAR	Not indicated (surveillance)
Recurrent and persistent pain			TEVAR	Not indicated (surveillance)
Large aortic diameter (≥40 mm)			TEVAR	Not indicated (surveillance)

IRAD: International Registry of Acute Aortic Dissection; SVS: Society for Vascular Surgery; STS: Society of Thoracic Surgeons; TEVAR: thoracic endovascular aortic repair; EVT: endovascular treatment

Since the indications for invasive treatment of acute and subacute type B dissection have changed significantly since 2000, we have divided the indications into two categories to avoid confusion.

### Life-saving invasive treatment for narrowly defined complicated cases

Rupture and abdominal/leg malperfusion are indications to save a patient’s life from acute aortic dissection. The treatment indications and proposes are the same as those before 2000, when TEVAR was not commonly used for type B dissection.

### Invasive treatment to improve the prognosis in the chronic phase for complicated cases in the broad sense newly added after 2000

Refractory hypertension (uncontrolled hypertension even with 3 antihypertensive drugs including beta blockers), recurrent and persistent pain, and large aortic diameter (≥40 mm) are the indications for invasive treatment of acute and subacute type B dissection, because the prognosis of these patients with conservative medical follow-up to the chronic phase is poor.^5^^–^^9^^,^^13^^–^^15)^ Thus, invasive treatment for these conditions has significant preemptive characteristics. These indications were added to the indications for invasive treatment after TEVAR was applied to aortic dissection.

As a side note, in “Reporting Standard for type B aortic dissociation” reported jointly by the Society for Vascular Surgery (SVS)/Society of Thoracic Surgeons (STS) in 2020, the indicated case in 2 is classified as an (uncomplicated) high-risk feature, not as a complicated case.^16)^ In addition to the above three conditions, five other conditions are listed as uncomplicated high-risk features: hematogenous pleural effusion, malperfusion on imaging, re-hospitalization, entry on the lesser curvature, and false lumen diameter >22 mm. Preemptive TEVAR is recommended for patients with uncomplicated high-risk features due to the predicted poor prognosis with continued conservative treatment.

## Timing and Method of Invasive Treatment (**Table 2**)

**Table:** Table 2 Interventions for type B aortic dissection by indication and timing

Disease stage (from onset)	Hyperacute phase (≤48 hr)	Acute phase (≤14 D)		Subacute phase (≤3 M)		Early chronic phase (<12 M)		Chronic phase (≥1 yr)
Indication	Malperfusion and rupture (impending rupture)	Refractory hypertension, recurrent and persistent thoracodorsal pain, and large aortic diameter (≥40 mm)			Hemorrhagic pleural effusion, malperfusion on imaging, re-hospitalization, entry on the lesser curvature side, false lumen diameter >22 mm			False lumen dilatation and aneurysm formation
Definition before 2000	Complicated	Uncomplicated						
IRAD definition (broad sense)	Complicated				Uncomplicated			
SVS/STS reporting standard	Complicated	(Uncomplicated) high-risk feature						N/A
Invasive treatment	1st: TEVAR (if TEVAR is not possible, then fenestration is permitted for malperfusion)	TEVAR						1st: graft replacement
	If endovascular treatment is not possible or ineffective, open surgery (graft replacement or fenestration)							If graft replacement is not possible or high risk, then TEVAR

IRAD: International Registry of Acute Aortic Dissection; SVS: Society for Vascular Surgery; STS: Society of Thoracic Surgeons; N/A: not applicable; TEVAR: thoracic endovascular aortic repair

The treatment methods differ according to the indication and stage of the disease. Complicated cases in the narrow sense (rupture, malperfusion) require emergency treatment in the hyperacute or acute phase and are mainly treated with TEVAR. For other broadly complicated cases or uncomplicated high-risk feature cases, TEVAR is performed in the subacute to early chronic phase (within 6 months of onset). In addition, for chronic cases of false lumen enlargement (dissecting aortic aneurysm) more than several years after the onset of dissection, surgery or TEVAR is performed depending on the disease and anatomy of the patient.

This section describes treatment methods for each pathological condition, focusing on invasive treatment in the acute, subacute, and early chronic phases.

### Rupture (impending rupture) cases (**Fig. 1A**)

**Figure 1:**
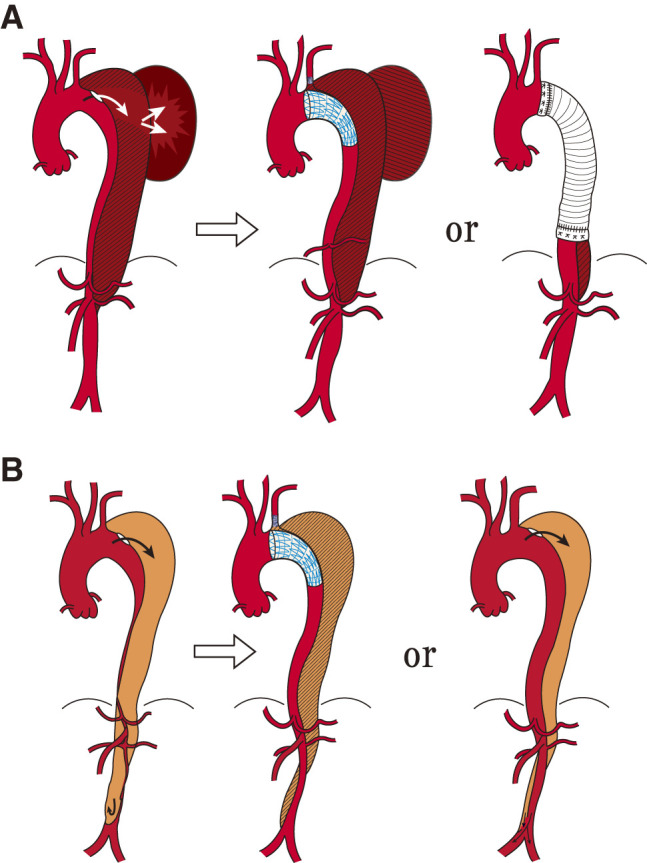
Fig. 1 (**A**) Emergent TEVAR or emergent open surgery for a rupture case with acute type B aortic dissection. (**B**) Emergent TEVAR or emergent open surgery for a malperfusion case with acute type B aortic dissection. TEVAR: thoracic endovascular aortic repair

When rupture is diagnosed based on symptoms (shock vitality or recovery from such a situation) and imaging (hematoma or high-density blood retention around the dissected aorta by computed tomography [CT], etc.), the patient is immediately transferred to a hybrid operating room where catheterization can be performed (in an emergency) after confirming the presence of the entry and its site. After transfer to the hybrid operating room, in patient with a single entry confirmed, TEVAR is performed to repair a sufficient extent around the entry site (at least 30 mm of landings at the proximal and distal to the entry). When the left subclavian artery and celiac artery are in the TEVAR graft extent, they must be covered. After confirming hemostasis by the TEVAR, revascularization should be considered for these covered branches.

When the entry cannot be found by any modality on preoperative imaging, extensive TEVAR from zone 2 or zone 3 to just above the celiac trunk should be performed.

When there are multiple entries and a patent false lumen, TEVAR is indicated only if all the multiple entries can be closed and the ruptured site of the false lumen can be completely excluded from the blood flow; otherwise, open surgery is required. In the open surgery, the ruptured area is replaced with an artificial graft. In such cases, assisted circulation with cardiopulmonary support is required after lateral thoracotomy, and intraoperative circulatory control, including preoperative vital management, is a major problem. If total bypass is obtained by cannulation into the ascending aorta or arch branch, hemodynamic stability is easily achieved. It is not life-saving unless the facility is experienced in lateral thoracotomy, and even if the patient’s life is saved, complications such as paraplegia are likely to occur.

### Abdominal and lower extremities malperfusion cases (**Fig. 1B**)

The type, range, and cause of malperfusion must be diagnosed based on symptoms (e.g., abdominal pain, lumbar pain, lower limb pain, poor palpation of the lower body pulse, and paraplegia) and diagnostic imaging (e.g., contrast-enhanced CT and abdominal vascular ultrasound). After these are ascertained, the patient is transferred to the hybrid operating room as an emergent case. Malperfusion is classified as a dynamic obstruction, mixed obstruction, and static obstruction based on the mechanism of the malperfusion; closure of the major entry by TEVAR is the most effective treatment for dynamic obstruction, and if it is difficult to achieve TEVAR, fenestration (transcatheter or surgical fenestration) on the abdominal aorta is performed. When it is difficult to perform TEVAR because the entry is located in the arch, total arch repair using the frozen elephant trunk technique will be performed in some cases. Axillofemoral bypass, which has been widely used in Japan for cases with malperfusion of lower extremity does not solve the cause of dynamic obstruction (inflow into the false lumen >>outflow into the true lumen) that occurs with dissection and should be considered a temporary treatment option when TEVAR and fenestration are not possible.

For mixed obstruction, in addition to the aortic treatments for dynamic obstruction (TEVAR and fenestration), direct treatment to the responsible branch vessel is needed, and transcatheter recanalization and stenting or bypass surgery are performed. Theoretically, static obstruction can be solved by direct treatment to the responsible branch vessel alone (transcatheter stenting or bypass surgery); however, hemodynamics after revascularization requires extra attention, and central repair with TEVAR may be necessary.

Central repair by TEVAR and fenestration (endovascular treatment and surgery) are described in detail below.

#### Central repair by TEVAR (entry closure) and petticoat stenting

Entry closure by TEVAR fundamentally improves the mechanism of dynamic obstruction (blood flow entering the false lumen from the entry is difficult to return to the true lumen, resulting in increased pressure in the false lumen compress the true lumen) and is often effective even if entry closure is not perfectly achieved. It has also been reported that insertion of a bare stent (petticoat stent) into the true lumen distal to the stent graft after entry closure by TEVAR more effectively improves true lumen blood flow, and thrombosis and remodeling of the false lumen are good.^17^^–^^20)^ The petticoat stent is generally placed to overlap a 2–3-cm distal part of the stent graft and reach the aortic bifurcation.

#### Transcatheter fenestration (**Fig. 2A**)

**Figure 2:**
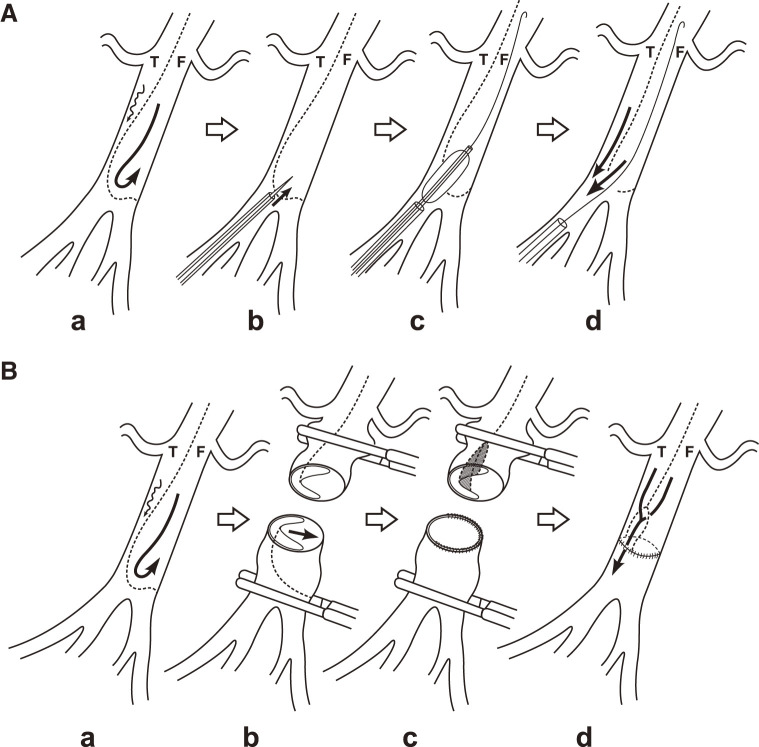
Fig. 2 (**A**) Schema of the transcatheter fenestration procedure. (**a**) The true lumen (T) is compressed by the dilated false lumen (F). (**b**) The dissecting septum is penetrated by a special needle that is inserted into the true lumen from the femoral artery, if necessary. (**c**) The angioplasty balloon is fully dilated and pulled across the dissected septum. (**d**) The true luminal narrowing and malperfusion of distal arteries are counteracted by the newly created reentry window. (**B**) Schema of the surgical fenestration procedure. (**a**) The true lumen (T) is compressed by the dilated false lumen (F). (**b**) The aorta is transected. (**c**) The proximal intima is resected and the distal aorta is reconstructed. (**d**) The aorta is re-sutured between the partially adventitial proximal aorta and the reconstructed distal aorta.

Access from the true lumen whenever possible, puncture the dissected intima at the level of the abdominal aorta below the renal artery using a Brockenbrough needle (Medtronic; Minneapolis, Minnesota, USA) or Rösch-Uchida needle (Cook Medical; Bloomington, Indiana, USA), pass the wire into the false lumen, and expand the punctured flap with a percutaneous transluminal angioplasty balloon. The balloon diameter must not exceed the vascular diameter at the same site.

#### Surgical fenestration (**Fig. 2B**)

Laparotomy and clamping at two sites of the aorta below the renal artery are performed. A transverse incision (or transection) is made around the true lumen, the flap is partially resected to create a fenestra, and suture closure of the transverse incision of the aorta.

### Preemptive TEVAR cases

In the acute and subacute phases, patients with refractory hypertension, recurrent and persistent pain, large aortic diameter (≥40 mm), accumulation of hemorrhagic pleural effusion, malperfusion on imaging, re-hospitalization, entry tear on the lesser curvature, and false lumen diameter >22 mm, TEVAR should be considered at either the acute–subacute phase or the early chronic phase (within 1 year of onset). The timing of treatment is urgent in symptomatic cases (e.g., refractory hypertension, recurrent or persistent pain), and preemptive TEVAR is performed in uncomplicated high-risk cases without symptoms. While closure of the primary entry alone by TEVAR is sufficient, all major entries located in the chest area are recommended to be closed, as long as it does not increase the risks. Small communication (intramural blood pooling) caused by a tear in the intercostal arteries is left untreated and follow-up observation is performed. Preemptive TEVAR should be performed with greater care to prevent complications, because there is no major risk if conservative treatment is continued instead of undergoing TEVAR.

Points of attention during treatment are as follows.

#### The range of stent-graft placement

Proximally, the portion without dissection is used as the landing zone. If the dissected portion can be avoided by choosing zone 2 landing, then debranching should be proactively performed and zone 2 landing should be selected. Although the distal side is often the dissected portion, the curved position should be avoided whenever possible. The length of the stent graft should be necessary and sufficient to close the target entry.

#### Sizing

In the proximal and distal landing areas, stent-graft diameter should be set at a size of 100%–120% of the true lumen diameter. The diameter of the lumen is used as the diameter of the deformed lumen when the lumen circumference is considered as a regular circle. In addition, since measurement of the true lumen is prone to inaccuracies in transverse and flexure sections, an aortic cross-sectional axial image perpendicular to the blood flow should be created for measurement. Due care should be paid to oversizing because it is a common cause of retrograde type A aortic dissection (RTAD) and stent graft-induced new entry tear (SINE) in the proximal and distal regions, respectively.

#### Device choice

There is often a difference in true lumen size between the proximal and distal landing areas of about 8–10 mm in the caliber. For this reason, the device should be selected tapered type accordingly, or two devices with different calibers should be stacked from the distal device. To avoid a bird beak in the proximal landing zone, devices that can be placed coaxially in the aorta should be planned and selected. Furthermore, the presence of a proximal bare stent, together with oversizing and bird beak mentioned above, is a factor in the occurrence of RTAD, so careful device selection is necessary.^21)^

## Mortality and Complications from Invasive Treatment

The early mortality rates of invasive treatment for type B dissection are summarized in **Table 3** by its indication.

**Table:** Table 3 Treatment outcomes for type B aortic dissection by indication

Indication	Procedure	Mortality	
Rupture	Graft replacement	14%–40%^22^^–^^24)^	17.5%–29.9%^12^^,^^25)^
	TEVAR	0%–25%^22^^,^^24^^,^^26^^,^^27)^	2.8%–13.3%^8^^,^^12^^,^^30^^–^^41)^
Malperfusion	TEVAR + EVT	7.7%–14.6%^26^^,^^28^^,^^29)^	
	Fenestration (open)		
	Fenestration (EVT)	7.7%^42)^	
Preemptive	TEVAR	2%–7.1%^43^^–^^46)^	

TEVAR: thoracic endovascular aortic repair; EVT: endovascular treatment

### Invasive treatment outcomes for rupture case

The outcome of treatment for rupture is the worst result of type B dissection invasive treatment in both TEVAR and open surgery. Even if the patient is saved in the operating room, severe systemic inflammatory response syndrome is likely to occur after treatment, and hypercytokinemia and acute respiratory distress syndrome should be noted. In TEVAR, there is also the possibility of re-rupture due to endoleak or other reasons, so strict management should be performed early in the postoperative period with these factors in mind. In addition, the incidence of spinal cord injury, which may be due to poor preoperative hemodynamics and disruption of collateral blood flow, is high, and additional procedures such as spinal drainage may be necessary immediately after the operation.

### Invasive treatment outcomes for malperfusion case

Malperfusion of superior mesenteric artery (SMA) carries a risk of intestinal necrosis even postoperatively and requires sensitivity to symptoms and abnormal laboratory examination. If strong symptoms persist, repeat ultrasonography and contrast-enhanced CT to closely examine the perfusion status, and do not hesitate to open the abdomen for an exploratory laparotomy. In addition, after treatment of extensive malperfusion, sever reperfusion injury such as myonephropathic metabolic syndrome may occur, and if myoglobinuria (urine occult blood: ++++, urine red cells: absent–small amount) is observed, appropriate action must be taken urgently.

### Invasive treatment outcomes for uncomplicated high-risk case

The worst complication of TEVAR for type B dissociation is RTAD. Especially in preemptive TEVAR, it is important to take great concern about RTAD and to plan to avoid it. **Table 4** summarizes risk factors for RTAD. Emergency surgery should not be hesitated for RTAD.

**Table:** Table 4 Risk factors for RTAD after TEVAR

Factor	RR	95% CI
Acute dissection (vs. chronic)	1.81	1.04–3.14
Dissection (vs. TAA)	5.33	2.70–10.51
Proximal bare stent (vs. non bare)	2.06	1.22–3.50
**Stent-graft oversizing**	**OR**	**P-value**
1% increase in oversizing >9%	1.14	<0.0001
**Proximal landing zone**	**Incidence (%)**	**P-value**
Zone 0	8.12	<0.0001
Zone 1	2.57	
Zone 2	2.66	
Zone 3 and 4	0.67	

RTAD: retrograde type A aortic dissection; TEVAR: thoracic endovascular aortic repair; RR: risk ratio; CI: confidence interval; OR: odds ratio; TAA: thoracic aortic aneurysm

Distal SINE after TEVAR is not a major problem when treated with additional TEVAR.

## Summary

The treatment of type B aortic dissection has undergone significant changes since the turn of the millennium with the introduction of TEVAR. It is necessary to closely monitor future trends, which may change significantly depending on the outcome of TEVAR treatment or the development of new treatment modalities.

## Additional Remark

This paper was presented at the 35th educational seminar at the Annual Meeting of the Japanese Society for Vascular Surgery (October 29, 2022, Yokohama).

## Disclosure Statement

The author has no conflicts of interest to declare.
